# 4D Flow Analysis of BAV-Related Fluid-Dynamic Alterations: Evidences of Wall Shear Stress Alterations in Absence of Clinically-Relevant Aortic Anatomical Remodeling

**DOI:** 10.3389/fphys.2017.00441

**Published:** 2017-06-26

**Authors:** Filippo Piatti, Francesco Sturla, Malenka M. Bissell, Selene Pirola, Massimo Lombardi, Igor Nesteruk, Alessandro Della Corte, Alberto C. L. Redaelli, Emiliano Votta

**Affiliations:** ^1^Department of Electronics, Information and Bioengineering, Politecnico di MilanoMilan, Italy; ^2^Division of Cardiovascular Medicine, Radcliffe Department of Medicine, University of OxfordOxford, United Kingdom; ^3^Department of Chemical Engineering, Imperial College LondonLondon, United Kingdom; ^4^Multimodality Cardiac Imaging Section, IRCCS Policlinico San DonatoSan Donato Milanese, Milan, Italy; ^5^Department of Free Boundary Flows, Institute of Hydromechanics, National Academy of Sciences of UkraineKyiv, Ukraine; ^6^Department of Cardiothoracic and Respiratory Sciences, Università Degli Studi Della Campania ‘L. Vanvitelli’ NaplesNaples, Italy

**Keywords:** bicuspid aortic valve, aortopathy, fluid dynamics, wall shear stress, magnetic resonance imaging, 4D flow

## Abstract

Bicuspid aortic valve (BAV) is the most common congenital cardiac disease and is a foremost risk factor for aortopathies. Despite the genetic basis of BAV and of the associated aortopathies, BAV-related alterations in aortic fluid-dynamics, and particularly in wall shear stresses (WSSs), likely play a role in the progression of aortopathy, and may contribute to its pathogenesis. To test whether WSS may trigger aortopathy, in this study we used 4D Flow sequences of phase-contrast cardiac magnetic resonance imaging (CMR) to quantitatively compare the *in vivo* fluid dynamics in the thoracic aorta of two groups of subjects: (i) five prospectively enrolled young patients with normo-functional BAV and with no aortic dilation and (ii) ten age-matched healthy volunteers. Through the semi-automated processing of 4D Flow data, the aortic bulk flow at peak systole was quantified, and WSSs acting on the endothelium of the ascending aorta were characterized throughout the systolic phase in terms of magnitude and time-dependency through a method recently developed by our group. Variables computed for each BAV patient were compared vs. the corresponding distribution of values obtained for healthy controls. In BAV patients, ascending aorta diameter was measured on cine-CMR images at baseline and at 3-year follow-up. As compared to controls, normo-functional BAV patients were characterized by minor bulk flow disturbances at peak systole. However, they were characterized by evident alterations of WSS distribution and peak values in the ascending aorta. In particular, in four BAV patients, who were characterized by right-left leaflet fusion, WSS peak values exceeded by 27–46% the 90th percentile of the distribution obtained for healthy volunteers. Only in the BAV patient with right-non-coronary leaflet fusion the same threshold was exceeded by 132%. Also, evident alterations in the time-dependency of WSS magnitude and direction were observed. Despite, these fluid-dynamic alterations, no clinically relevant anatomical remodeling was observed in the BAV patients at 3-year follow-up. In light of previous evidence from the literature, our results suggest that WSS alterations may precede the onset of aortopathy and may contribute to its triggering, but WSS-driven anatomical remodeling, if any, is a very slow process.

## Introduction

Bicuspid aortic valve (BAV) is the most common congenital cardiac disease and affects about 2% of newborns (Michelena et al., 2014). BAV is a recognized risk factor for aortopathies, with more than 50% of BAV patients developing aneurysm of the ascending aorta (Nistri et al., 1999; Fedak et al., 2002).

The development of BAV-related aortopathies has been attributed to genetic and hemodynamic bases (Verma and Siu, 2014), with several studies focusing on their relative contribution (Fedak et al., 2002; Siu and Silversides, 2010; Girdauskas et al., 2011; Wendell et al., 2013; Guzzardi et al., 2015).

In regard to fluid dynamics, the velocity jet during systole has been shown to be deflected in the BAV aorta (Robicsek et al., 2004; Della Corte et al., 2012). This alteration causes different nested helical flow patterns in the ascending aorta depending on the BAV fusion phenotype (Hope et al., 2010), as well as a regional abnormal distribution of wall shear stress (WSS) stimuli on the aortic wall (Bissell et al., 2013). Through mechanotransduction pathways, these alterations may trigger altered gene and protein expression, inflammatory processes, and altered regulation of endothelial and smooth muscle cells (Chien, 2006; Chiu and Chien, 2011; Wang et al., 2013; McCormick et al., 2017). Indeed, increased WSS stimuli are associated with extracellular matrix (ECM) dysregulation and elastic fiber degeneration (Guzzardi et al., 2015; Albinsson et al., 2017). Regions with elevated WSS revealed an increase in the concentration of transforming growth factor (TGF)-β1 and activation of aortic wall matrix metalloproteinase (MMP): TGF-β1 is strongly implicated in the mechanotransduction of WSS upstream of flow-induced vascular remodeling (Ohno et al., 1995), while MMP is highly implicated in degradation of elastic ECM components (Chung et al., 2007). Interestingly, differential expression of MicroRNAs, which can modulate mechanotransduction pathways, was detected in the convexity and concavity of the ascending aorta of BAV patients with mild aortic dilation, suggesting a potential role of fluid-dynamics already at the early stage of aortopathy (Albinsson et al., 2017).

To better clarify the potential role of BAV-related aortic flow alterations in the onset and development of aortopathies, recent studies isolated the effects of such alterations in non-dilated aortas through computational fluid dynamics (CFD) and *in vitro* experiments. Cao et al. (2017) used CFD to quantify the WSS acting on the wall of a non-dilated aorta in presence of healthy aortic valve and of BAV with different fusion patterns. That analysis highlighted the alterations in terms of WSS magnitude and time-oscillations induced by the different BAV morphotypes. McNally et al. (2017) achieved similar results through *in vitro* experiments, in which two-dimensional particle image velocimetry (PIV) was used to quantify the velocity distribution and the shear stress distribution in the non-dilated ascending aorta associated to different BAV fusion patterns. In an elegant study (Atkins et al., 2014), integrated the numerical and *in vitro* approaches. Through CFD, the authors computed the highly space- and time-resolved distribution of WSS over the convexity of the aorta in presence of healthy aortic valve and BAV, respectively, and extracted the time-course of WSS at relevant spots. Based on those data, samples of healthy tissue from the extrados of porcine aortas were stimulated for 48 h in highly controlled conditions with healthy and BAV-related WSS histories. In the latter case, the authors observed an increase in the expression of MMPs involved in the degradation of the aortic wall *tunica media*. Although these studies provided relevant evidences, they suffered from some limitations. As BAV-related fluid-dynamics was quantified through CFD, the simplifying assumptions typically characterizing this approach were adopted (e.g., rigid aortic wall and idealized velocity profiles used as inlet boundary conditions). Also, computations were performed with paradigmatic configurations of the thoracic aorta and of the aortic valve.

Recently, 3D time-resolved phase-contrast cardiac magnetic resonance pulse sequences with three-directional velocity-encoding (4D Flow) have been used to direct quantify the *in vivo* fluid dynamics in the aorta, and proved to be a valuable technique to elucidate the role of BAV-related WSS alterations (Bissell et al., 2013; Hope et al., 2014) on a patient-specific basis. In principle, this approach could allow for overcoming the limitations of computational and *in vitro* modeling approaches. Yet, from a technical standpoint, the reliability of WSS computation is challenged by technical limits of 4D Flow sequences, specifically in terms of spatial and temporal resolution of the measured discrete velocity data (Markl et al., 2014). State of the art sequences typically obtain isotropic voxels with a characteristic dimension ranging from 1.5 to 2 mm, while the cardiac cycle is sampled in 20–30 phases, resulting in a temporal resolution of 30–50 ms (Dyverfeldt et al., 2015). Indeed, reliable descriptions of WSS spatial distributions and good reproducibility in WSS estimations were found when comparing BAV patients against control groups (Barker et al., 2012a; Bissell et al., 2013; Van Ooij et al., 2014a), supporting the fact that WSS estimation may have a role as a tool to grade the level of aortopathy progression. Nevertheless, the aforementioned limitations of 4D Flow sequences hamper the accuracy of WSS computations, which have been proved to underestimate expected *in vivo* values up to one order of magnitude (Petersson et al., 2012; Piatti et al., 2016). To reduce the impact of these limitations, we recently developed a novel method for the quantification of WSSs that was proved to be more accurate as compared to the previous state of the art (Piatti et al., 2016). Also, from a clinical standpoint, published studies typically focused on BAV patients with pre-existing aortic remodeling and aortic valve stenosis (Barker et al., 2012b; Hope et al., 2014; Van Ooij et al., 2014b). These derangements may impact on the quantified fluid dynamic features, making them unsuitable to gain insight on the specific role of WSS in the onset of BAV-related aortopathies.

To test the possible role of WSS alterations as triggers of aortopathy, in the present work we used 4D Flow data to quantify the *in vivo* fluid dynamics and WSS distributions in the thoracic aorta of young normo-functional BAV patients with no clinical evidence of aortic stenosis or aortic wall remodeling, and we quantitatively compared results vs. a cohort of healthy controls.

## Materials and methods

### Clinical population and follow-up

Five patients with a normo-functional BAV were recruited prospectively at a single university hospital from cardiology clinics, cardiac MRI, and echocardiography sessions. Detailed demographics of the enrolled patients are reported in Table 1. 2D cine-MRI images were used to analyze valve function and measure aortic diameters on double oblique planes positioned along the aortic root. Aortic diameter was measured next to the right pulmonary artery. This measurement was repeated at 3-year follow-up to assess potential aortic remodeling. Ten age-matched healthy volunteers (HV, female males, age of 23 ± 7) were enrolled as control group and were free from any known significant medical problem. Both BAV patients and HVs underwent 4D Flow acquisitions. The outcome of follow-up measurements on BAV patients was not known when 4D Flow data were processed. The study complies with the declaration of Helsinki and was approved by the West Berkshire ethics committee, United Kingdom. All participants gave written informed consent.

**Table 1:** Demographics of BAV patients.

	**BAV patients (*n* = 5)**
Age [years]	25 ± 10
Sex (Male:Female)	4:1
BSA [m^2^]	1.83 ± 0.123
Lesion Cusps (RL:RN:LN)	4:1:0
AI (Mild:Moderate:Severe)	1:0:0
AS (Mild:Moderate:Severe)	4:1:0
AAo [mm]	24.2 ± 2.7
AAo/BSA [mm/m^2^]	13.3 ± 2.1
Hear rate [bpm]	61 ± 5
Cardiac Output [l/min]	5.2 ± 1.0
Peripheral Diastolic Blood Pressure [mmHg]	58 ± 7
Peripheral Systolic Blood Pressure [mmHg]	113 ± 8
Mean Arterial Pressure [mmHg]	76 ± 9
**COMORBIDITIES**	
Cardiovascular problems	none
Hay fever	2
Heart burn	1
Gastritis	1

*BAV, Bicuspid aortic valve; BSA, body surface area; RL, right-left fusion; RN, right and non-coronary fusion; LN, left and non-coronary fusion; AI, aortic insufficiency; AS, aortic stenosis; AAo, ascending aorta diameter. Values are displayed as mean ± SD*.

### 4D flow acquisitions

4D flow acquisitions were performed at John Radcliffe Hospital (Oxford, United Kingdom) on a 3.0 T MR system (Trio, Siemens, Erlangen, Germany). Flow-sensitive gradient-echo pulse sequence MRI was used to characterize and quantify blood flow in the thoracic aorta. Datasets were acquired with prospective ECG-gating during free-breathing, using a respiratory navigator. The volume of acquisition was oriented along an oblique-sagittal plane encompassing the ascending aorta, the aortic arch, and the thoracic aorta. 4D Flow sequences were performed with the following parameters: echo time = 2.29–2.63 ms, repetition time = 38.4–41.6 ms, flip angle = 7°, voxel size = 1.46–1.67 × 1.46–1.67 × 1.8–2.2 mm^3^, temporal resolution = 50.72–76.91 ms. The velocity-encoding range (VENC) was properly determined using the lowest non-aliasing velocity on scout measurements, resulting in 1.3–1.5 m/s and 1.5–1.9 m/s for HVs and BAV patients, respectively.

### 4D flow data processing

Through *ad-hoc* in-house software implemented in MATLAB (The MathWorks Inc., Natick, MA, United States), 4D Flow data were analyzed through a semi-automated pipeline that consisted of five steps.

**Figure 1:**
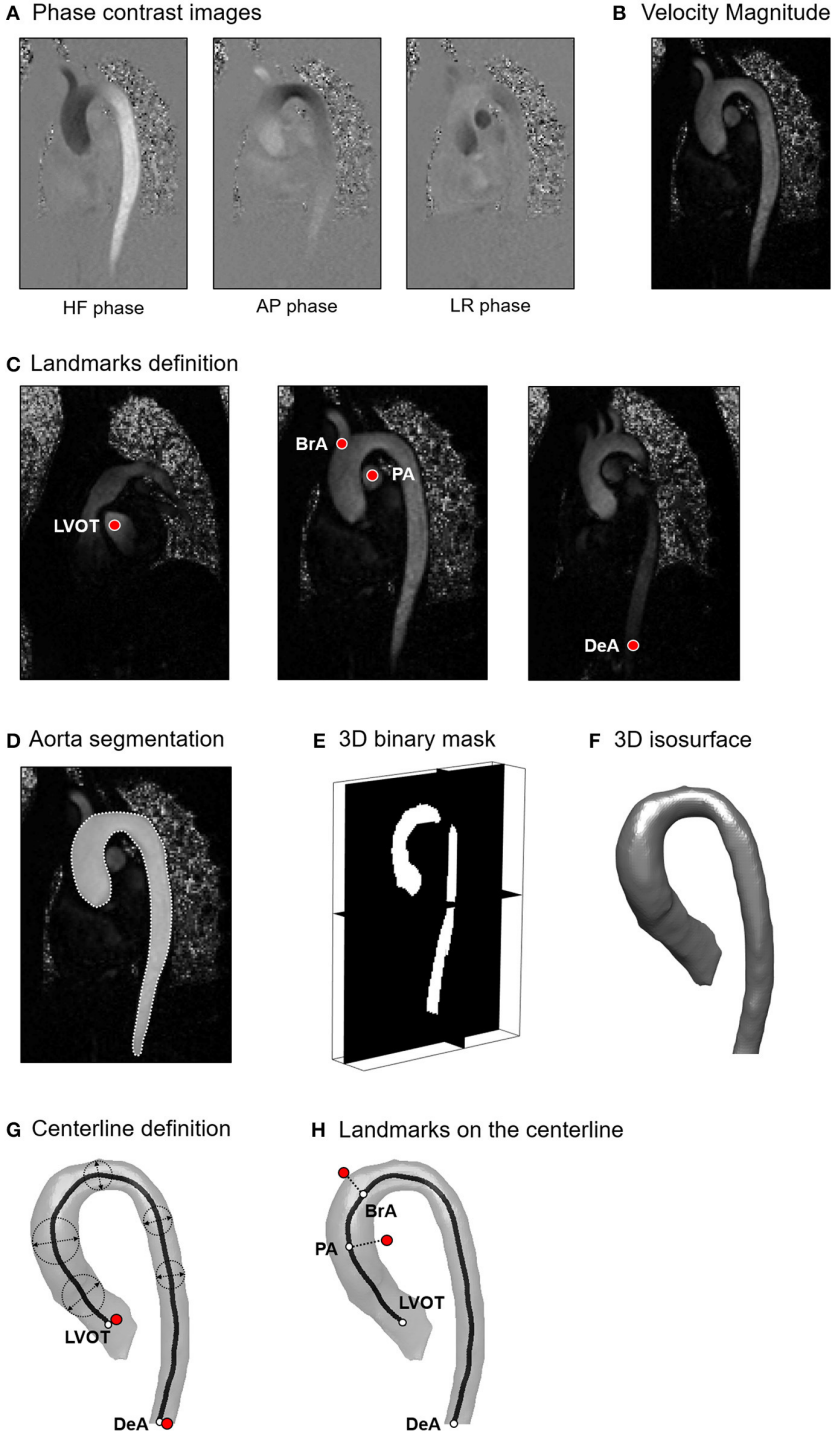
**(A)** Phase contrast images encoded for Head-Foot (HF), Antero-Posterior (AP) and Left-Right (LR) directions; **(B)** Magnitude intensity of the velocity vector computed as the Euclidean norm of the 3D velocity vectors. **(C)** Definition of anatomically relevant landmarks of the aortic lumen: left ventricle outflow tract (LVOT), brachiocephalic artery (BrA), right pulmonary artery (PA), distal point of the descending aorta (DeA); **(D)** Tracing of the aortic lumen boundaries; **(E)** 3D binary mask obtained from the stack of manually traced slices; **(F)** 3D isosurface of the aortic lumen; **(G)** Computation of the vessel centerline; **(H)** Projection of the identified landmarks on the centerline.

The first step consisted of 4D flow velocity data encoding and pre-filtering. Phase-contrast images of each encoded flow direction (Figure 1A) were scaled by the corresponding VENC value to obtain the Cartesian velocity components (Markl et al., 2011). Velocity components were automatically processed to correct from eddy current errors, velocity aliasing and to decrease MR noise through median filtering (Bock et al., 2007). The Euclidean norm of the 3D velocity vectors was then computed based on the Cartesian components for all acquired time frames to obtain the magnitude intensity of the velocity vector (Figure 1B).

In the second step, through visual inspection of the dataset, the time-frame with the highest velocity-to-noise ratio was identified as the most representative of peak systole. At the selected time-frame, four landmarks were manually positioned inside the acquired volume at locations corresponding to the left ventricle outflow tract (LVOT), right pulmonary artery (PA), brachiocephalic artery (BrA), and the most distal visible point of the descending aorta (DeA; Figure 1C). Moreover, the 3D geometry of the aortic lumen was defined. The boundaries of the lumen were manually traced to define the region of interest (ROI) on each para-sagittal slice of the 4D Flow volume (Figure 1D). Tracing resulted in a 2D binary mask associating a value equal to 1 to the voxels inside the traced boundary, and equal to 0 otherwise. A 3D binary mask was obtained merging the 2D masks (Figure 1E). Subsequently, the aortic wall and the centerline of the aorta were automatically defined. The wall was identified as the closed iso-surface encompassing the borders of the 3D binary ROI, and it was smoothed through Laplacian diffusion filtering (Desbrun et al., 1999) to obtain a regularized surface mesh (Figure 1F). The centerline of the vessel wall was obtained through skeletonization of the ROI (Shih, 2010); the points defining the skeleton were ordered end-to-end via Dijkstra algorithm (Dijkstra, 1959), specifying the projections of LVOT and DeA landmarks on the skeleton as starting and ending points, respectively (Figure 1G). The resulting ordered points were fitted with non-uniform rational bi-cubic spline (NURBS) curve interpolation that was smoothed via 5th order moving average filtering to obtain the centerline of the vessel wall. PA and BrA landmarks were finally projected to the closest points on the centerline (Figure 1H).

**Figure 2:**
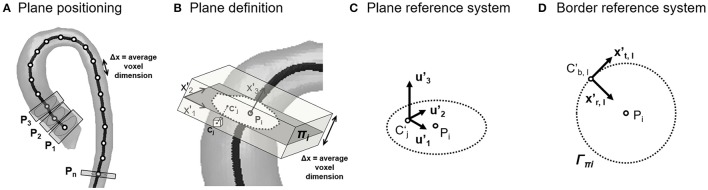
**(A)** Position along the centerline of the points (P_i_) used to define the cross-sections of the aortic lumen; **(B)** On each cross-section, original discrete velocity points (C_j_) enclosed by the segmentation and within a tolerance (0.51 of a voxel) were projected as (C'_j_) on the plane (π_i_) and interpolated. According to the orientation of the cross-section, local reference systems were defined inside the vessel lumen **(C)** and on the vessel's wall **(D)**.

In the third step, aorta cross-sections were defined along the vessel's centerline. Starting from LVOT, a set of equally spaced points **P**_i_ was automatically generated on the vessel centerline, prescribing a distance Δx between consecutive points **P**_i_ and **P**_i+1_ equal to the average voxel dimension of the dataset (Figure 2A). Each point **P**_i_ and its corresponding unit vector **x'**_3_ locally tangent to the centerline were used to automatically define a cross-section π_i_ passing through **P**_i_ and normal to **x'**_3_ (Figure 2B). For each voxel whose center **C**_j_ was located within 0.5^*^Δx from π_i_, the projection (**C'**_j_) of **C**_j_ was computed (Figure 2B), and the three velocity components (**u**_1j_, **u**_2j_, **u**_3j_) measured at **C**_j_ were associated to **C'**_j_. The discrete velocity values obtained over π_i_ were interpolated through cubic splines to increase the spatial resolution of data, and velocity components were transformed into the local reference system (**x'**_1_**, x'**_2_**, x'**_3_**;** Figure 2C). In addition, for each point **C'**_B,l_ of π_i_ belonging to the boundary of the vessel wall Γ_π_, a second local reference system was defined, which consisted of the local inward normal to the wall surface, assumed as the radial direction **x'**_r,l_, the normal to the cross section **x'**_3_, assumed as the longitudinal direction, and the cross product of **x'**_r,l_ by **x'**_3_, assumed as the circumferential direction **x'**_t,l_ (Figure 2D).

In the fourth step, the aortic bulk flow was automatically quantified. Velocity data of each cross-section were processed to compute the variables of interest:

- Vessel cross-sectional area A_*i*_, as the surface of the piecewise linear polygon defined by the boundary points **C'_B,l_**;
- Equivalent diameter $D_{i}=2\cdot \sqrt{A_{i}}/\pi$;
- Net flow rate waveform *Q*_*i*_(*t*). At each time-frame *t*_*k*_, $Q_{i}\left(t_{k}\right)\;=\;\frac{A_{i}}{N_{i}}\sum_{1}^{N}v\prime 3\;\left(t_{k}\right)$, where *N*_*i*_ is the number of data points over the i-th cross section. The time-frames corresponding to peak systole (T_Ps_) and to the end of systole (T_Es_) were automatically identified based on *Q*_*i*_(*t*), as the maximum point and as the last point of the descending phase of *Q*_*i*_(*t*), respectively. The mid time-frame in the T_Ps_ − T_Es_ transitory was considered as the mid-deceleration time point (T_dec_);
- Peak flow rate $\left(Q_{max}^{LVOT}\right)$ and stroke volume (SV) at the level of the LVOT. SV was estimated as the area under *Q*_*i*_(*t*), via trapezoidal method between the first time-frame, i.e., the onset of systole, and T_Es_;
- Peak and mean values of velocity magnitudes (**|V|**_**i_max**_ and **|V|**_**i_mean**_, respectively);
- Flow jet angle (α_πi_), defined as the angle between the mean positive velocity vector, i.e., the average of vectors with positive component *v*′_3_, and **x'**_3_ (Sigovan et al., 2011);
- Normalized flow displacement (**d**_πi_), defined as the distance between the center of velocity and the center of the lumen **P**_i_ (Sigovan et al., 2011).

**|V|**_**i_max**_, **|V|**_**i_mean**_, α_πi_ and **d**_πi_ were computed at three consecutive time-frames centered in T_ps_. The analysis was focused on two specific sectors of the ascending aorta: the proximal tract, running from LVOT to PA landmarks, and the distal tract, running from PA to BrA.

The values of the listed hemodynamic variables computed in BAV patients were compared off-line vs. those computed for HVs with non-parametric Mann–Whitney *t*-test, adopting a *p* = 0.05 as statistically significant, using GraphPad Prism 7 (GraphPad Software, Inc., La Jolla, CA, USA).

In the fifth step, the distribution of WSS vectors ($\vec{WSS}$) acting on the aortic endothelium was quantified. To this aim, the stresses tensor τ_*ij*_ was computed at the boundary of the fluid domain under the assumption of Newtonian blood rheological behavior (Baskurt and Meiselman, 2003):

$$\tau_{ij}=\mu \left(\frac{\partial u_{i}}{\partial x_{j}}+\frac{\partial u_{j}}{\partial x_{i}}\right)\;\;with\;\;i\neq j$$

where viscosity μ was set to 3.7cP (Baskurt and Meiselman, 2003), and spatial derivatives of velocities were computed numerically. In the local reference system **x'**_r,l_, **x'**_t,l_, **x'**_3_, where **x'**_r,l_ is the local inward normal to the aortic wall, the 3D vector **T** was computed as:

$$T=\tau \;\cdot \;x_{r,l}^{\prime }$$

The 3D shear stress vector $\vec{WSS}$ was then computed as:

$$\vec{WSS}=x_{r,l}^{\prime }\times \left(T\times x_{r,l}^{\prime }\right)$$

The axial (${\vec{WSS}}_{Axial}$) and circumferential (${\vec{WSS}}_{Circ}$) components of $\vec{WSS}$ were finally obtained by projecting $\vec{WSS}$ on the local axes **x'**_**3**_ and **x'**_t,l_, respectively.

$\vec{WSS}$ was computed throughout the systolic phase, i.e., from the first time-frame to **T_Es_**. As in a recent work of our group (Piatti et al., 2016), the numerical computation of the spatial derivatives of velocity was performed through two novel methods.

The first method, named Global Volumetric (GV), is based on the use of 3D Sobel filters to compute voxelwise the components of the strain rate tensor and yielded the distribution of ${\vec{WSS}}_{GV}$ for each point of the triangular elements of the surface representing the aortic wall. We showed previously that this method correctly captures the spatial trends in $\vec{WSS}$, despite underestimating pointwise values (Piatti et al., 2016). Additionally, the oscillations in time of ${\vec{WSS}}_{GV}$ were computed by means of the Oscillatory Shear Index (OSI), of the Relative Residence Time (RRT), and of the Transversal WSS (TransWSS), as defined in Table 2 (Soulis et al., 2011; Jahangiri et al., 2015; Mohamied et al., 2017). Specifically, the OSI quantifies changes in $\vec{WSS}$ direction during time, and ranges from 0.0 to 0.5 as $\vec{WSS}$ rotates by 0° to 180°. The OSI was computed also for the single components ${\vec{WSS}}_{Axial}$ and ${\vec{WSS}}_{Circ}$, obtaining the two indexes OSI_Ax_ and OSI_Circ_ (Supplementary Material). RRT combines the effect of time-averaged WSS vector magnitude at a given region of the endothelium with its corresponding OSI value: RRT gets lower as $\vec{WSS}$ increases and OSI decreases, i.e., when locally the wall is loaded by fast and constant flow jets, and it gets greater as $\vec{WSS}$ decreases and OSI increases, i.e., when the wall is loaded by slow and highly time-dependent flow jets. TransWSS measures the component of the $\vec{WSS}$ that is perpendicular to the time-averaged $\vec{WSS}$, representative of the average direction of the flow. This metric is hence defined in a reference frame based on the flow direction and not on the geometry of the vessel wall.

**Table 2:** Definition of time-averaged WSS magnitude (AWSS), time-averaged WSS vector (AWSSV), Oscillatory Shear Index (OSI), Relative Residence Time (RRT), and Transversal WSS (TransWSS).

**Variable**	**Definition**
AWSS [Pa]	$\frac{1}{T}\int_{0}^{T}|\vec{WSS}|dt$
AWSSV [Pa]	$\frac{1}{T}|\int_{0}^{T}\vec{WSS}dt|$
OSI [−]	$0.5\cdot \left(1-\frac{AWSSV}{AWSS}\right)$
RRT [Pa^−1^]	[(1−2.0·*OSI*)·*AWSS*]^−1^
TransWSS [Pa]	$|\vec{WSS}|\cdot \left(\vec{n}\wedge \frac{\int_{0}^{T}\vec{WSS}dt}{\left|\int_{0}^{T}\vec{WSS}dt\right|}\right)$

The second method, named Local Planar (LP), was adopted to obtain the distribution of ${\vec{WSS}}_{LP}$ for specific locations of the vessel wall based on the velocity values over a single vessel cross-section. As demonstrated in Piatti et al. (2016), this approach allows for improving the pointwise quantification of $\vec{WSS}$. It is based on the application of 2D derivative filters; in Piatti et al. (2016), 2D Sobel filters were exploited to compute the components of the strain rate tensor, while in the present study B-Spline based filters (Hast, 2014) were used, owing to the improved results verified through preliminary benchmarking (unpublished data). ${\vec{WSS}}_{LP}$ was characterized in terms of magnitude and angle that quantifies the deviation of ${\vec{WSS}}_{LP}$ from the longitudinal, i.e., axial, direction of the vessel (for a 0° angle, the WSS vector is purely axial and points downstream from the considered cross-section; for 90° angle, the WSS vector is purely circumferential; for a 180° angle, the WSS vector is again purely axial, but points upstream of the considered cross-section).

### Representation of results at the aortic wall

**Figure 3:**
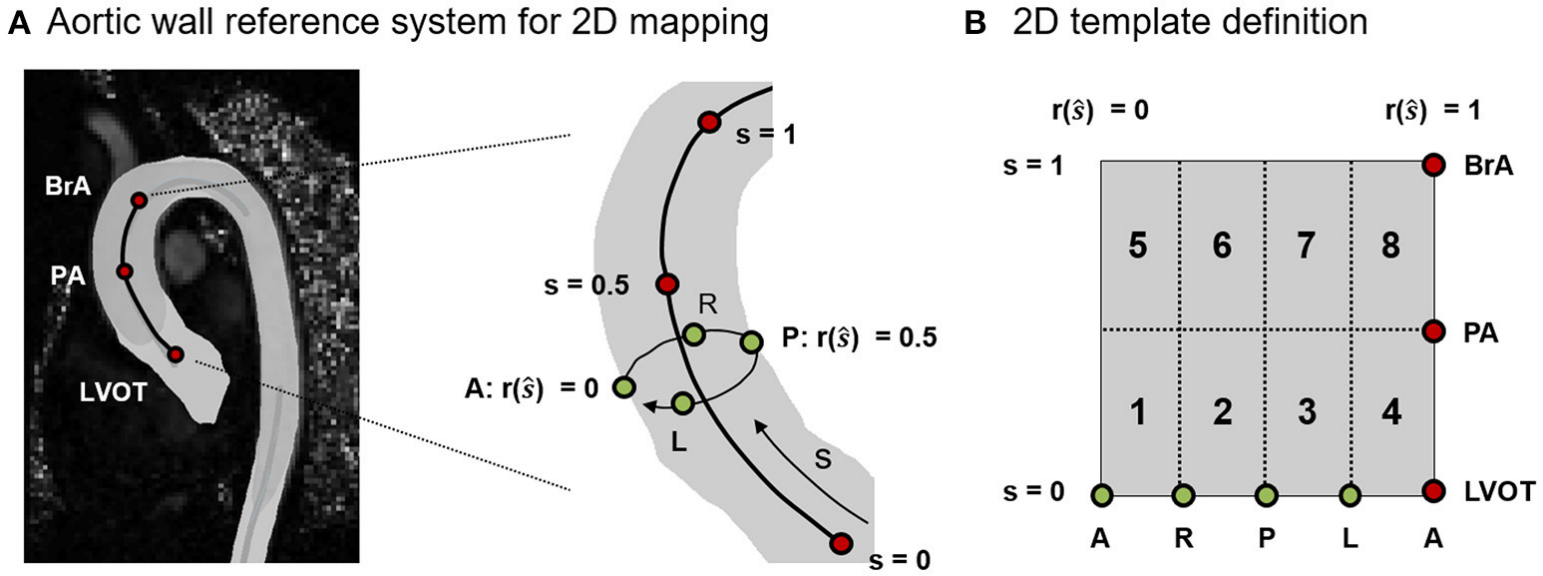
**(A)** Definition of the normalized reference system used to map on a 2D template the spatial localization of points on the 3D aortic wall. The longitudinal position of points is represented by s (*s* = 0 at LVOT and *s* = 1 at BrA). For a given cross section at *s* = ŝ, the circumferential position is represented by r(ŝ). **(B)** Sub-division of the 2D template into 8 sectors based on the anatomical landmarks (LVOT, PA, BrA) and orientation points (Antero: A, Posterior: P, Left: L, Right: R).

The analysis of ${\vec{WSS}}_{GV}$, OSI and RRT data was focused on the ascending aorta, i.e., on the tract of the vessel running from LVOT to BrA. ${\vec{WSS}}_{GV}$ values were averaged over three time-frames centered in **T_ps_**. To rule out the effects of inter-subject anatomical variability when comparing different aortas, ${\vec{WSS}}_{GV}$, OSI and RRT data were represented in terms of heat maps over a normalized 2D template (Figure 3A). The position of points in the relevant tract of the aortic wall was expressed in terms of: (i) normalized longitudinal position *s* along the centerline with *s* = 0 at LVOT and *s* = 1 at BrA; (ii) given a cross section at *s* = ŝ, normalized circumferential position *r*(ŝ) along the boundary of the cross-section (*r* = 0 at the most anterior point, and increases when moving counterclockwise over the boundary). The 2D template was sub-divided into 8 sectors with respect to LVOT, PA and BrA landmarks and anatomical short axis orientations (Antero: A; Posterior: P; Right: R; Left: L; Figure 3B).

## Results

### Aortic growth at 3-year follow up

No clinically relevant increase in diameter was measured at 3-year follow-up. On patients BAV02-BAV05, an increase in diameter by <1.5 mm was measured, which is below the threshold indicated by guidelines as symptom of disease progression (0.5 mm/year, Della Corte et al., 2013). On patient BAV01, an increase in diameter by 1.8 mm was measured, which was equivalent to a yearly growth rate of 0.6 mm/year, only slightly above the threshold. However, 1.8 mm is comparable to the in-plane resolution of the cine-MRI images used to perform the measurements, and indeed no macroscopic remodeling of the aorta could be observed.

### Aorta bulk flow

**Figure 4:**
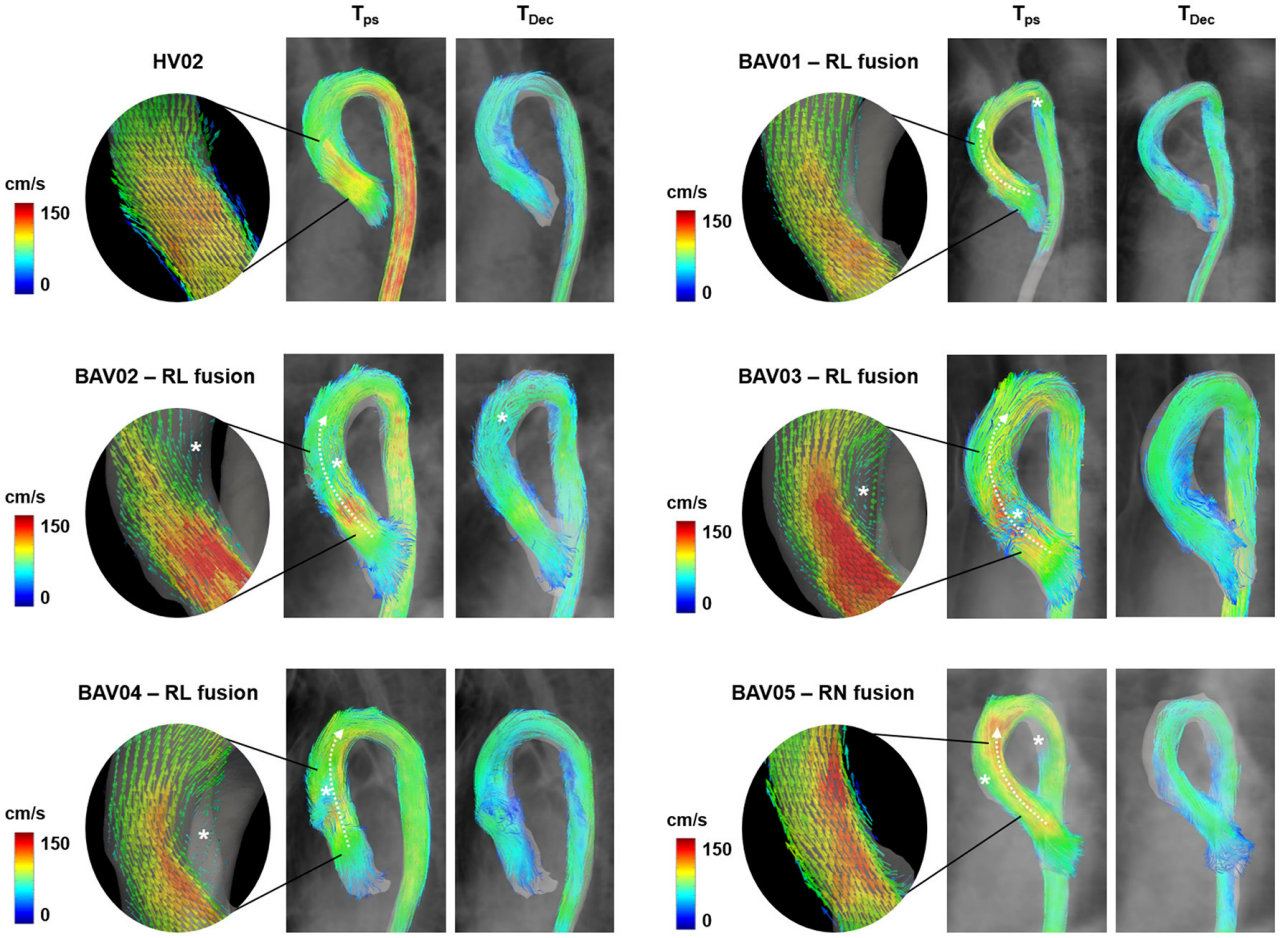
Visualization of 3D blood flow streamlines within the aortic lumen at the time-frames corresponding to peak-systole (T_ps_) and to mid-deceleration phase (T_Dec_). In rounded panels, the features of blood flow jet across the aortic valve during systolic ejection are highlighted through vectors projected on a long-axis view of the left ventricle outflow tract. White asterisks are reported in presence of macroscopic altered secondary flows.

For all the analyzed datasets, streamlines over the whole acquired domain within the ROI were computed and visualized using ParaView (Sandia Corporation, Kitware Inc., Albuquerque, NM, USA) at *t* = T_ps_ and *t* = T_dec_ (Figure 4).

Qualitatively, HVs were systematically characterized by a centered and nearly laminar flow in the entire ROI, as exemplified by dataset HV02 (Figure 4). At T_ps_, macroscopic secondary flows were absent, but were observed at T_dec_ close to the intrados of the ascending aorta. All normo-BAV patients presented altered secondary flows at T_ps_, owing to the BAV deflected jet (Figure 4, white asterisks). The jet was directed anteriorly, toward the extrados of the aorta, in patients characterized by RL leaflet fusion (BAV01-BAV04). The jet was directed posteriorly, toward the intrados, in case of RN fusion (BAV05). Also, aberrant flows were observed in the distal curvature of the aortic arch in BAV01 and BAV05 patients. At T_dec_, the same pattern of secondary flows observed in HVs was observed also in BAV01, BAV03, and BAV04. The pattern was somewhat complementary, with disturbances displaced toward the extrados of the ascending aorta, in the only case of RN fusion (BAV05). More evident flow disturbances represented by helicoidal structures were observed all over the ascending aorta and arch in BAV02.

In BAV patients $Q_{max}^{LVOT}$ and SV values ranged from 12.6 to 26.3 l/min and from 80 to 156 ml/beat, respectively. These values were slightly higher as compared to those obtained for HVs, which ranged from 18.4 to 30.3 l/min and from 62 to 135 ml/beat, respectively. However, these differences were not statistically significant (*p* = 0.44 and *p* = 0.68, respectively).

For HVs and BAV patients, the peak value of **|V| _i_max_** and D_i_ and the average value of **|V|_i_mean_**, **α_πi_** and **d**_πi_ over the centerline of the ascending aorta were considered for the two tracts LVOT-PA and PA-BrA (Table 3). No statistically significant differences were observed between the two groups in terms of peak D_i_ in the LVOT–PA tract (*p* = 0.31) nor in the PA–BrA tract (*p* = 0.25). Statistically significant differences between HVs and BAVs (*p* = 0.04) were found only for the peak values of **|V|_i_max_** in the LVOT–PA tract, and for the average value of **α_πi_** in the PA–BrA tract. Non-negligible though not statistically significant differences were observed for the average value of **|V|_i_mean_** in both tracts (*p* = *0.05* in the LVOT-PA tract, *p* = 0.07 in the PA-BrA tract), and for the peak value of **|V|_i_max_** (*p* = 0.05) in the PA–BrA tract.

**Table 3:** Hemodynamic variables computed along the centerline for HV and BAV patients, from LVOT to PA and from PA to BrA landmarks.

	**LVOT-PA**			**PA-BrA**		
	**HV**	**BAV**	***p***	**HV**	**BAV**	***p***
D_max_ [mm]	29.9 ÷ 20.5	28.8 ÷ 23.4	0.31	28.9 ÷ 20.4	29.0 ÷ 19.4	0.25
|V| _max_ [cm/s]	179.7 ÷ 116.4	204.6 ÷ 137.0	**0.04**	146.1 ÷ 84.6	205.5 ÷ 117.6	0.05
|V|_mean_ [cm/s]	97.1 ÷ 56.5	117.8 ÷ 77.9	0.05	82.1 ÷ 53.6	107.9 ÷ 64.0	0.07
d_π_ [−]	0.12 ÷ 0.04	0.07 ÷ 0.06	0.79	0.08 ÷ 0.03	0.09 ÷ 0.03	0.91
α_π_ [°]	12.8 ÷ 6.7	14.3 ÷ 7.4	0.77	21.8 ÷ 9.2	13.0 ÷ 7.6	**0.04**

*Statistically significant differences (p < 0.05) are reported in bold*.

### Fluid-dynamic stimuli on the aortic wall

**Figure 5:**
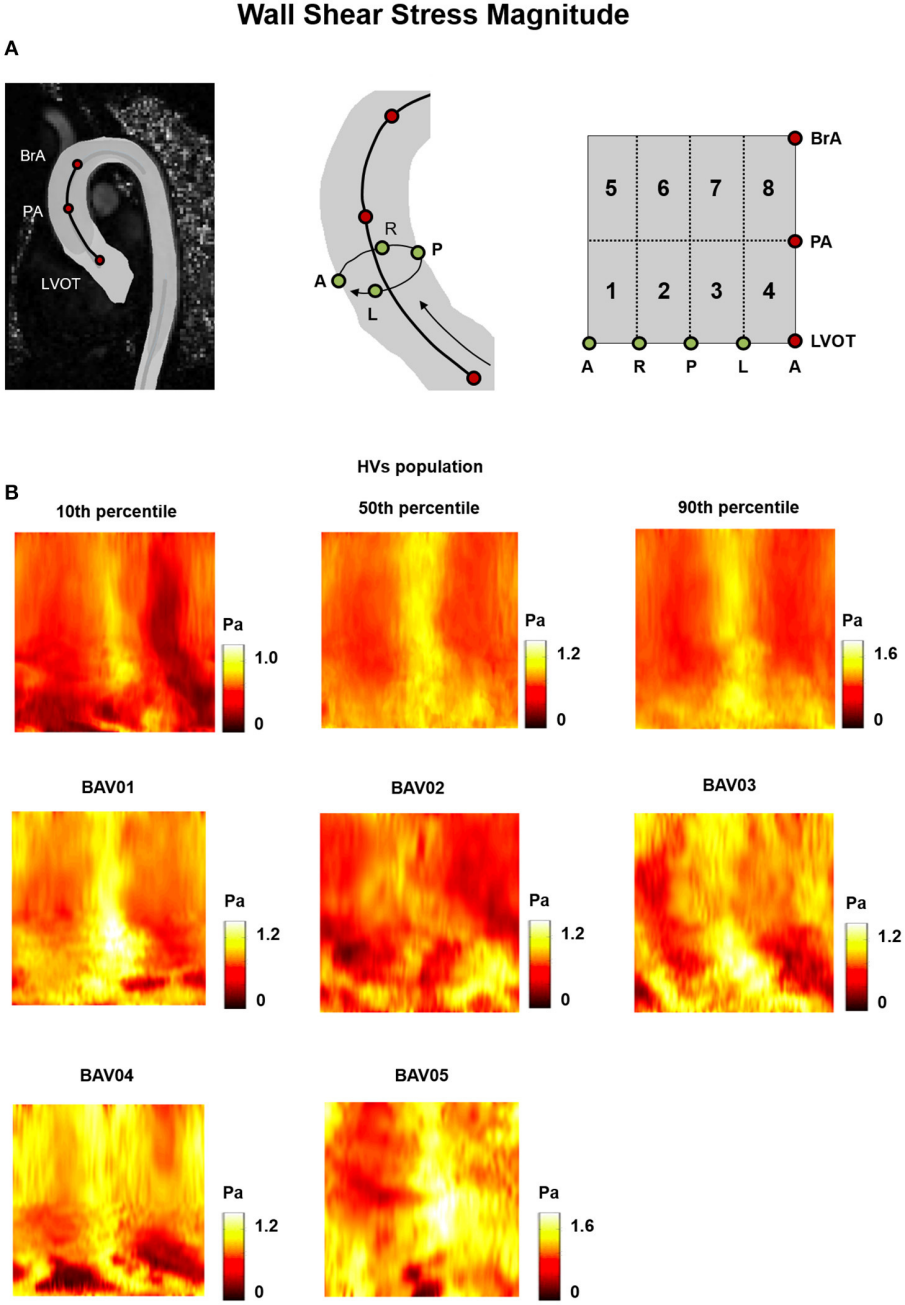
**(A)** The 2D template detailed in Figure 3 is reported for the sake of clarity. **(B)** Wall Shear Stress (WSS) heat maps for HVs (represented as 10th, 50th, and 90th percentiles) and for each BAV patient obtained at peak systole.

**Figure 6:**
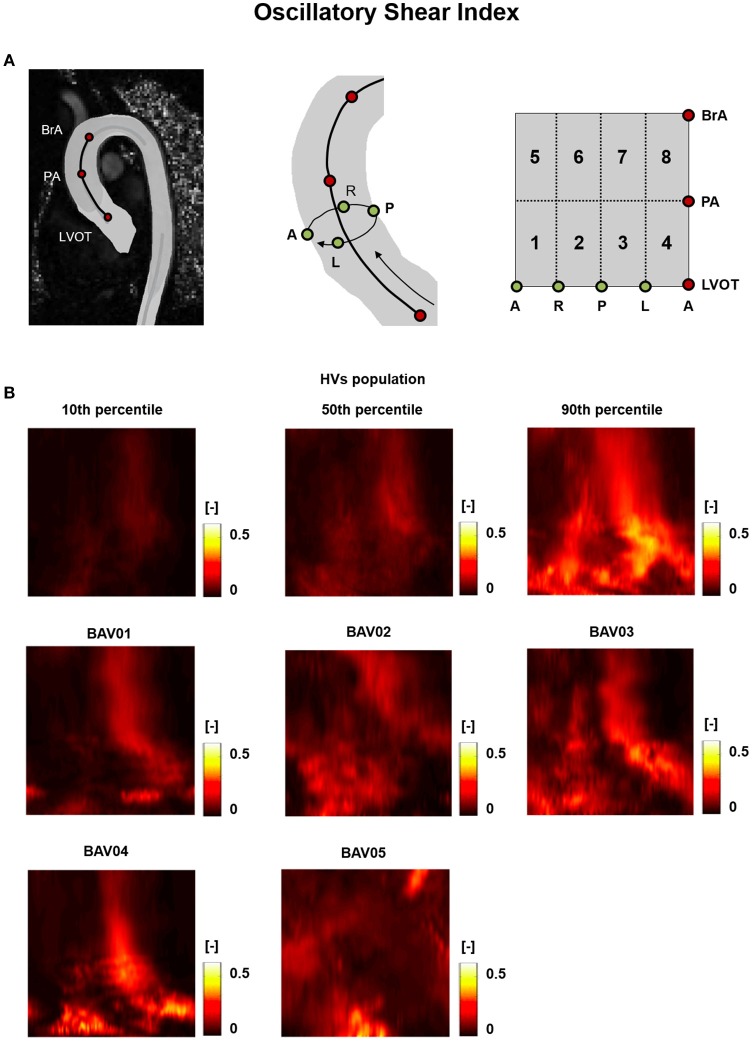
**(A)** The 2D template detailed in Figure 3 is reported for the sake of clarity. **(B)** Oscillatory Shear Index (OSI) heat maps for HVs (represented as 10th, 50th, and 90th percentiles) and for each BAV patient obtained through time-averaging over the systolic phase.

**Figure 7:**
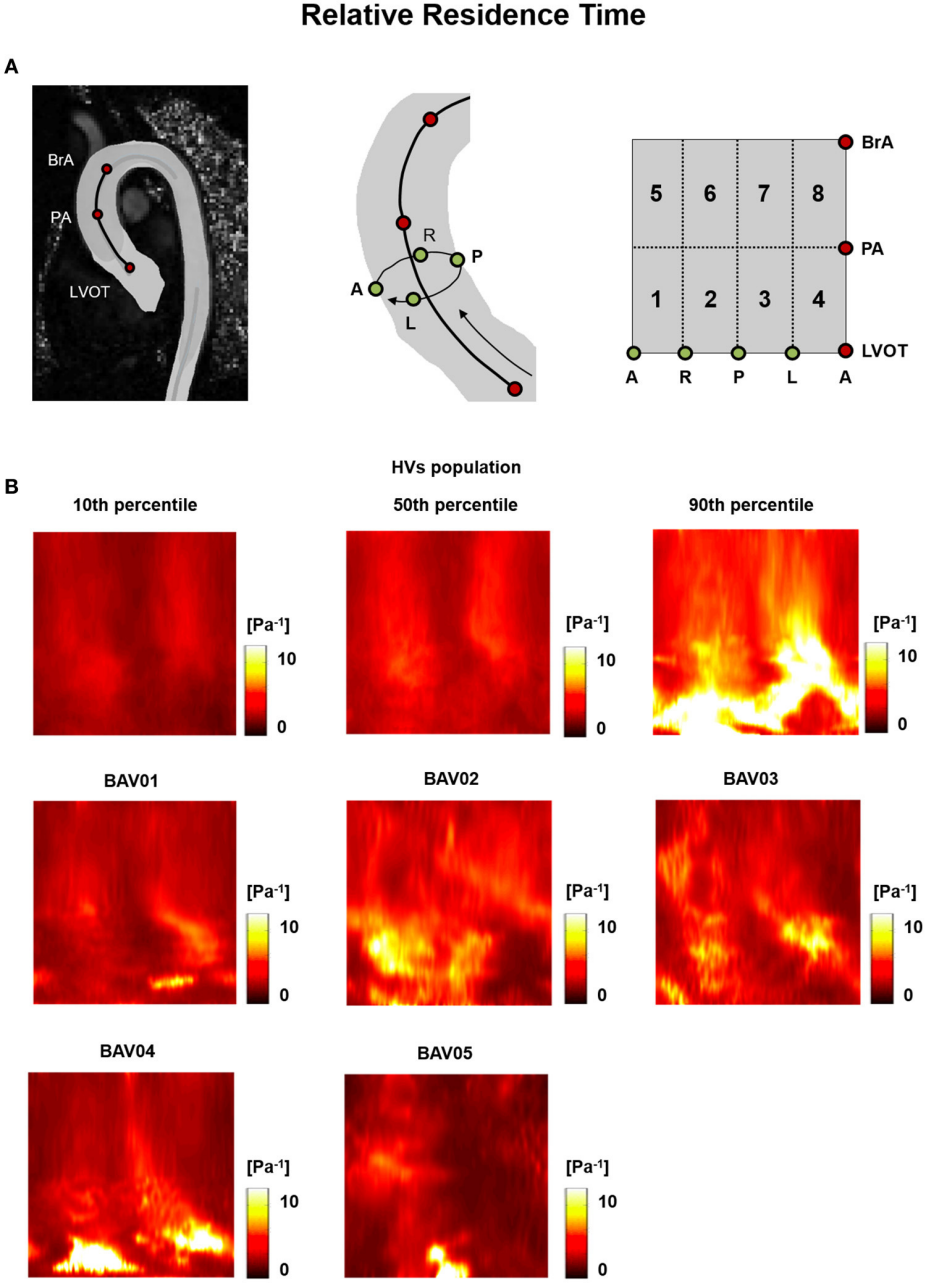
**(A)** The 2D template detailed in Figure 3 is reported for the sake of clarity. **(B)** Relative Residence Time (RRT) heat maps for HVs (represented as 10th, 50th, and 90th percentiles) and for each BAV patient obtained through time-averaging over the systolic phase.

For HVs, the heat maps of the 10th, 50^th^, and 90th percentiles of the $\left|{\vec{WSS}}_{GV}\right|$, OSI and RRT distribution in this group (Figures 5A, 6A, 7A) were used as term of comparison for the heat maps obtained for each BAV patient (Figures 5B, 6B, 7B).

In the HVs population, $\left|{\vec{WSS}}_{GV}\right|$ values were higher in the posterior and anterior sides of the wall of the distal ascending aorta, i.e., from PA up to BrA (posterior: 1.08 ÷ 1.13 Pa, anterior 0.90 ÷ 1.22 Pa), while lower values were detected in the lateral sides (right: 0.58 ÷ 0.80 Pa, left: 0.40 ÷ 0.56 Pa (Figure 5A). In the proximal ascending aorta (i.e., sectors immediately downstream of LVOT), $\left|{\vec{WSS}}_{GV}\right|$ was uniformly redistributed over the circumferential extent of the vessel wall, but with a high inter-subject variability (range: 0.20 ÷ 1.19 Pa), as underscored by the differences between 10th and 90th percentile heat maps. In BAV patients, a high inter-subject variability of $\left|{\vec{WSS}}_{GV}\right|$ distribution was evident all over the wall of the ascending aorta, with different patient-specific localizations and extent of high and low $\left|{\vec{WSS}}_{GV}\right|$ regions (Figure 5B).

For both HVs and BAV patients, the distribution of OSI was consistent with the one of $\left|{\vec{WSS}}_{GV}\right|$. For instance, in HVs the 50th percentile heat map showed regions of high OSI at the right and left side of the distal ascending aorta (right: 0.24 ÷ 0.38, left: 0.21 ÷ 0.28; Figure 6A), which corresponded to low WSS regions. A similar correspondence between low $\left|{\vec{WSS}}_{GV}\right|$ regions and high OSI values could be observed for most locations of the heat maps obtained for BAV patients, thus preserving a high inter-subject variability in the distribution of OSI values (Figure 6B). The decomposition of the OSI along the circumferential and axial directions of the vessel showed that in BAV patients extended regions of the aortic wall were characterized by OSI_*Ax*_ values exceeding the 90th percentile of the distribution observed in HVs (Supplementary Figure 1). These regions were consistent with those characterized by extremely high value of the OSI computed for the full WSS vector (Figure 6B). By contrast, in BAV patients wide regions of the aortic wall were characterized by OSI_*Circ*_ values that were below the 10th percentile of the distribution observed in HVs (Supplementary Figure 2).

RRT heat maps consistently combined the features observed for OSI and $\left|{\vec{WSS}}_{GV}\right|$ (Figure 7).

**Figure 8:**
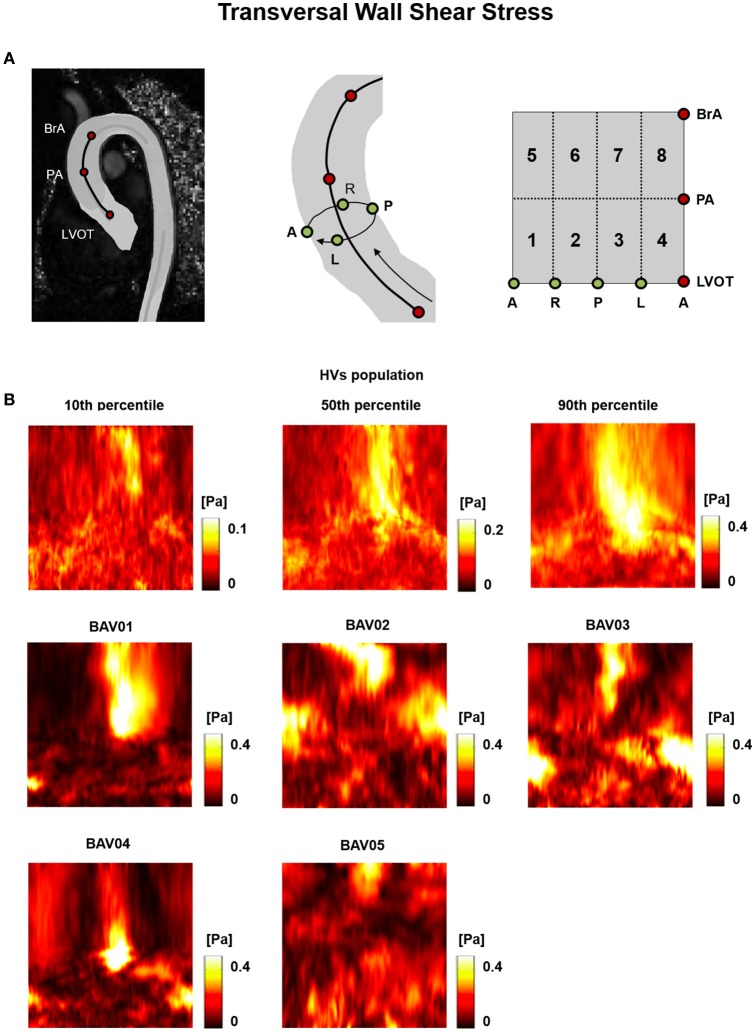
**(A)** The 2D template detailed in Figure 3 is reported for the sake of clarity. **(B)** Transversal WSS (TransWSS) heat maps for HVs (represented as 10th, 50th, and 90th percentiles) and for each BAV patient obtained at peak systole.

Differently from the previously reported metrics, the TransWSS was characterized by a lower inter-subject variability over the HVs: the only exception consisted in the posterior-left region, where TransWSS ranged from 0.06 to 0.39 Pa (Figure 8). Also, all BAV patients were characterized by spread regions with abnormally high or low TransWSSs. These differences were observed particularly in the distal ascending aorta, i.e., from PA up to BrA, with values ranging from 0.03 Pa to 0.51 Pa (Figure 8).

**Figure 9:**
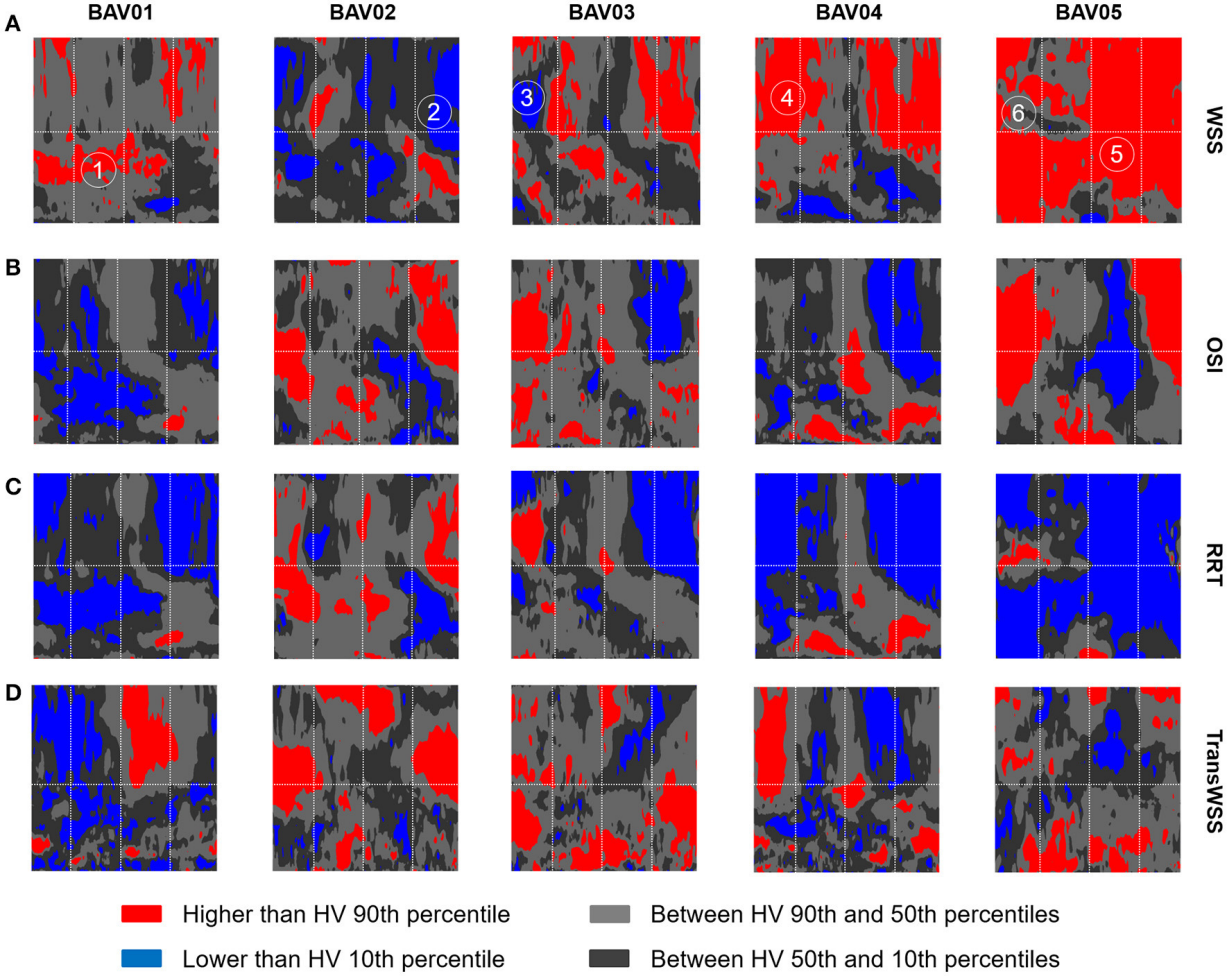
Representation on the 2D template detailed in Figure 3 of the HV-relative heat maps of WSS **(A)**, OSI **(B)**, RRT **(C)**, and TransWSS **(D)** computed for each BAV patient. The HV-relative heat maps are obtained from point-wise comparison of each BAV patient against HVs population and color-coded accordingly: higher than 90th percentile (red), lower than 10th percentile (blue), between 90th and 10th percentiles (light gray, dark gray). On the HV-relative heat maps, six hot-spots were labeled and identified.

To better highlight the patient-specific deviations of BAV patients from the HVs distribution, HV-relative heat maps were also obtained and represented on the same 2D template for each wall-related variable, with an approach resembling the one used in Van Ooij et al. (2014b). Specifically, each point on the wall of the ascending aorta was classified based on whether the corresponding value of the relevant variable was (i) abnormally high, i.e., higher than HVs 90th percentile, (ii) abnormally low, i.e., lower than HVs 10th percentile, (iii) between HVs 50th and 90th percentiles, (iv) between HVs 10th and 50th percentiles (Figure 9).

When considering BAV patients with RL fusion (BAV01-BAV04), localized regions with abnormally high $\left|{\vec{WSS}}_{GV}\right|$ values were detected for patient BAV02. Wide regions with abnormally high $\left|{\vec{WSS}}_{GV}\right|$ were detected on the distal ascending aorta of BAV03 and BAV04, and on the proximal ascending aorta of BAV01. Detected values exceeded the HV 90th percentile by up to 27.3, 27.3, 30.5, 45.8% for BAV01-BAV04, respectively. Regions of abnormally low $\left|{\vec{WSS}}_{GV}\right|$ were localized distally in BAV03 or proximally in BAV04, as well as in large portions of the anterior and posterior ascending aorta of BAV02. For patient BAV05, characterized by RN fusion, a pervasive abnormal increase in $\left|{\vec{WSS}}_{GV}\right|$ was evident: around 72% of the wall of the ascending aorta was subject to abnormally high $\left|{\vec{WSS}}_{GV}\right|$ values, which exceeded the HV 90th percentile by up to 131.7% (Figure 9A).

Regions characterized by abnormally high OSI values were found in the anterior sectors of the ascending aorta of patients from the RL fusion cohort (BAV02, BAV03) and for the patient with RN fusion (BAV05). In BAV04, few regions of abnormally high OSI values were found proximally, while abnormally low OSI values were computed in the distal left-extrados of the aortic wall. Of note, BAV01 was free from abnormally high OSI values, but was characterized by abnormally low OSI values over 22% of the ascending aorta wall (Figure 9B). Overall, RRT HV-relative heat maps showed a complementary pattern as compared to the obtained for $\left|{\vec{WSS}}_{GV}\right|$ values, thus confirming the prevalent contribution of $\left|{\vec{WSS}}_{GV}\right|$ over OSI in determining the value of RRT. This predominant behavior of $\left|{\vec{WSS}}_{GV}\right|$ was particularly evident in the anterior regions of the ascending aorta of BAV05 characterized by high values of both $\left|{\vec{WSS}}_{GV}\right|$ and OSI (Figure 9C). Considering the HV-relative maps of TransWSS computed at peak systole, BAV patients were characterized by ubiquitous differences with respect to the HVs population, both in regions above HV 90th percentile and below HV 10th percentile. These differences appeared more widely spread on the aortic wall for all BAV patients as compared to the patterns obtained for the other computed indexes (Figure 9D).

**Figure 10:**
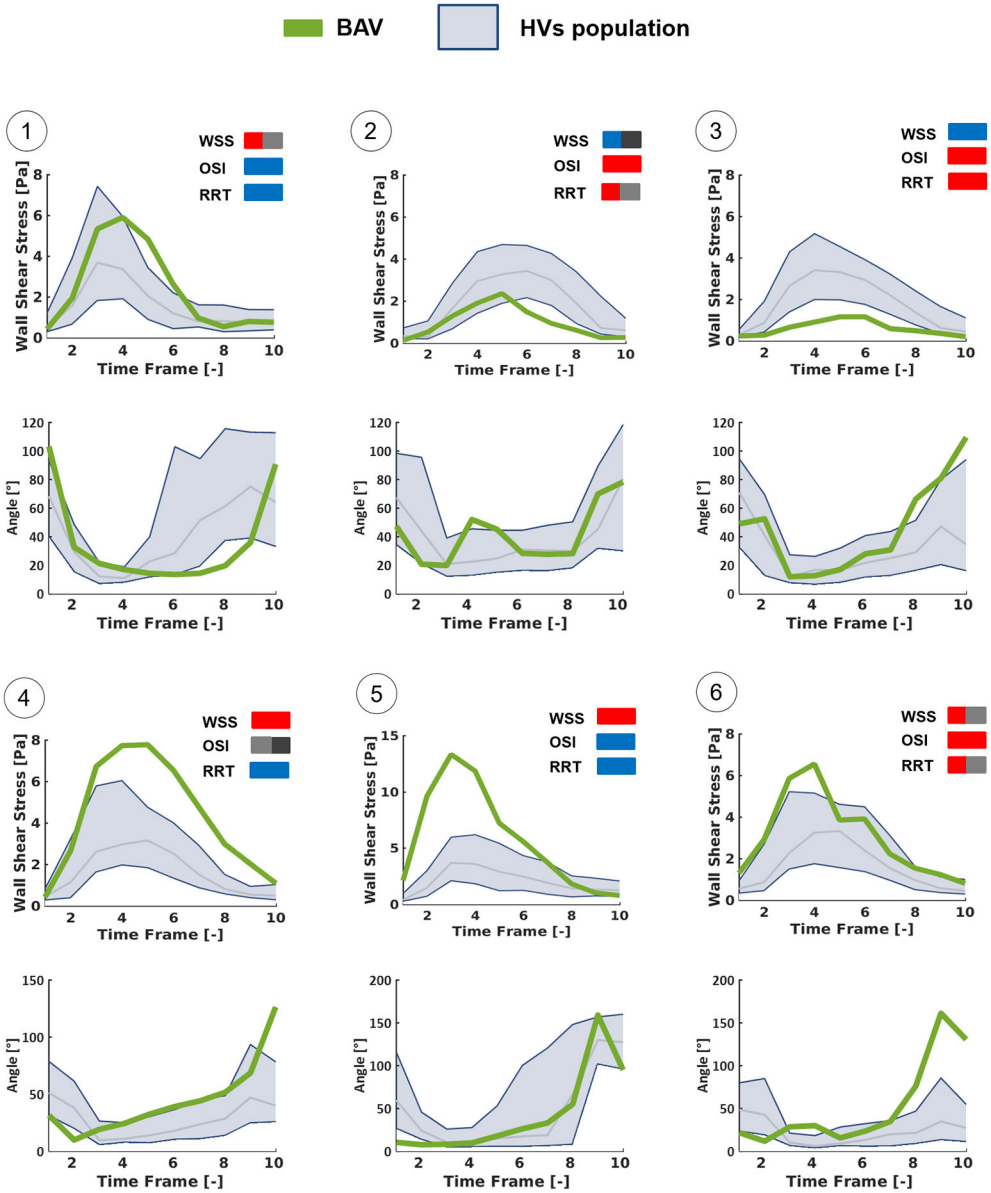
Time-dependent WSS waveforms computed at the selected hot-spots detailed in Figure 9 using the LP method, throughout the systolic phase. Each BAV patient-specific case (green line) was compared with HVs population (blue zone: from 10th to 90th percentiles) in terms of the magnitude of WSS vector and of the angle between it and the axial direction of the vessel.

Furthermore, six different hot-spots were selected (Figure 9A) on HV-relative $\left|{\vec{WSS}}_{GV}\right|$ heat maps of different BAV patients. These hot spots were selected because they were characterized by different combinations of $\left|{\vec{WSS}}_{GV}\right|$, OSI and RRT HV-relative classifications. At each selected hot spot, the time-course of magnitude and orientation of ${\vec{WSS}}_{LP}$ was computed. For both features, the time-course was compared to the time dependent range (10th–90th percentile) obtained in HVs at the same normalized location (Figure 10). Of note, computed $\left|{\vec{WSS}}_{LP}\right|$ values were approximately one order of magnitude higher than the $\left|{\vec{WSS}}_{GV}\right|$ computed over the aortic wall. This result is consistent with the much better capability of the LP approach, as compared to the GV one, to realistically capture $\left|\vec{WSS}\right|$ values, as demonstrated in Piatti et al. (2016). The analysis of the different time-courses of $\left|{\vec{WSS}}_{LP}\right|$ showed that regions of abnormally high values can be subject to overloading throughout the whole systolic phase (hot spots 4 and 5) or only for a part of it (hot spots 1 and 6). Considering the time-dependent courses of ${\vec{WSS}}_{LP}$ angle, similar ranges were found in BAV patients with respect to the corresponding hot-spots in HVs, especially considering the first part of the systole. However, the dynamics of angle variations showed altered patterns when associated either with high (hot spots 2, 3, and 6) or low (hot spot 1) OSI values. Also, hot spots characterized by abnormally high OSI values were systematically characterized also by time-oscillations of $\left|{\vec{WSS}}_{LP}\right|$ only during the systolic deceleration phase (hot spots 2, 3, and 6).

## Discussion

### Novelty of the study

In this work, we exploited the *in vivo* measurements yielded by 4D Flow sequences to analyze blood fluid dynamics and $\vec{WSS}$ -related indexes in young normo-functional BAV patients (age 25 ± 10 years) without aortic stenosis or aortic dilation and to compare them vs. age-matched healthy controls. To the best of our knowledge, this is the first study of this kind specifically focused on a cohort of BAV-affected patients without any anatomical derangement of the aorta at the organ length-scale. From this standpoint, the present study differs from previous studies based on 4D Flow imaging, which were run on very heterogeneous cohorts of BAV patients that included elder patients and subjects already affected by aortic dilation or aortic stenosis (Barker et al., 2012a; Hope et al., 2014). Consequently, those studies typically aimed to testing possible correlations between the altered fluid-dynamic stimuli on the aortic wall and its microstructural/anatomical derangements (Barker et al., 2012a; Bissell et al., 2013; Hope et al., 2014; Mahadevia et al., 2014) following the onset of aortopathy: differently, the present study aims at gaining insight into the possible role of such altered fluid-dynamic stimuli as trigger of the response pathways that ultimately lead to aortopathy.

A second novel aspect of the study resides in the computation of $\vec{WSS}$ and of the related metrics. Regarding the computation of $\vec{WSS}$, we obtained the 3D vector based on the calculation of the strain rate tensor through derivative filters. To the best of our knowledge, this method is not used by other research groups in the context of 4D Flow data processing. As expected from our previous benchmarking (Piatti et al., 2016), this method, and namely its implementation on 2D cross-sections, yielded values of $\vec{WSS}$ magnitude that are greater than those reported in the literature by approximately one order of magnitude. For instance, in Barker et al. (2012a) systolic values of 0.3 ± 0.1 Pa are reported for the ascending aorta of healthy subjects with a cardiac output of 5.2 ± 1.2 l/min, and values ranging from 0.4 ± 0.2 Pa to 0.8 ± 0.3 Pa, depending on the aortic side, are reported for BAV patients with non-stenotic valve, moderate aortic dilation, and cardiac output of 6.3 ± 1.7 l/min. Similarly, in Hope et al. (2011), even when considering the wall region impacted by highly eccentric systolic jets, peak $\vec{WSS}$ magnitude values of 1.67 Pa were reported in BAV patients. Regarding the $\vec{WSS}$ -related metrics, we provided for the first time such a broad spectrum of indexes, in the attempt to test whether some indexes may be more relevant in the context of BAV-related aortopathies.

### $\vec{wss}$-related indexes as a mean to detect altered fluid dynamics

In the present study, we could find remarkable abnormalities in the space distribution, in the peak magnitude values, and in the time-course of $\vec{WSS}$ acting on the ascending aorta of the considered cohort of BAV patients characterized by normo-functional valves and non-dilated aortas. These abnormalities were even more evident when considering $\vec{WSS}$ -derived indexes; in particular, every considered BAV patient was characterized by pervasive alterations of the TransWSS and by broad regions with extremely low values of OSI_Circ_, indicating that alterations affect not only peak $\left|\vec{WSS}\right|$, but also the time-course and the directionality of $\vec{WSS}$. Of note, the computed $\vec{WSS}$ -related alterations vs. healthy controls were remarkable and more evident than those appearing from the qualitative and quantitative analysis of the bulk flow. Also, within the sub-group of RL fusion patients, the flow field pattern appeared repeatable, in particular at peak systole. Instead, $\left|\vec{WSS}\right|$, OSI and RRT heatmaps highlighted a non-negligible inter-subject variability. These evidences should be cautiously evaluated owing to the small number of considered BAV patients, however they suggest that the quantitative analysis of $\vec{WSS}$ -derived indexes may have a greater potential to discriminate between healthy and aberrant fluid dynamics, and possibly to grade the severity of the BAV-related fluid-dynamic alterations on a patient-specific basis as compared to the analysis of the bulk flow field.

### Relevance of our results to the understanding of the progression of BAV-related aortopathy

Even though we computed far greater values of $\left|\vec{WSS}\right|$ as compared to previous studies focused on the ascending aorta and based on the processing of 4D Flow data, the alterations we found in our BAV patients vs. HVs were consistent, in terms of percentage variations, with those obtained on more heterogenous cohorts of BAV patients (Hope et al., 2010; Bissell et al., 2013), as well as on BAV patients characterized by severe aortic dilation and selected for immediate aortic resection (Van Ooij et al., 2014a). Yet, measurements of ascending aorta diameter at 3-year follow-up did not reveal any clinically relevant aortic anatomical remodeling at the organ length scale, despite the anomalies in $\vec{WSS}$ detected at baseline on the endothelium of the aortic wall. This evidence was true not only when abnormally high $\left|\vec{WSS}\right|$ values affected limited regions and exceeded HVs values by up to 46% (BAV01-BAV04), but also when abnormally high $\left|\vec{WSS}\right|$ values affected the entire ascending aorta and were characterized by a striking 132% increase as compared to HVs values (BAV05).

Hence, in the context of BAV-related aortopathy our results, if analyzed *per*-*se*, may suggest two mutually exclusive interpretations. On the one hand, one may conclude that there is no cause-effect relationship between altered fluid-dynamic stimuli and onset of aortopathy. On the other hand, one may conclude that the anatomical remodeling of the aortic wall due to altered fluid-dynamic stimuli is a slow process that develops over decades and may not be detected in young patients.

However, the analysis of our results in light of the evidences of previous studies from the literature suggests that the first interpretation is extremely unlikely, whereas the second one might be realistic. Indeed, recent studies investigated the effects of BAV-related fluid-dynamic alterations in non-dilated and initially healthy aortas, isolating these alterations from possible concomitant pathophysiological factors (Atkins et al., 2014; Atkins, 2016; Cao et al., 2017; McNally et al., 2017). Morphotype-dependent flow alterations in the aorta proved to generate different mechanical stimuli on the aortic wall in terms of $\vec{WSS}$ magnitude and directionality (Cao et al., 2017). These BAV-specific mechanical stimuli were different on the convexity and on the concavity of the ascending aorta. Interestingly, when applied *in vitro* to fresh specimens of healthy porcine aortas, these two region-dependent stimuli induced different responses: only when reproducing the $\vec{WSS}$ pattern computed at the convexity significant alterations in the expression of MMP-2 and MMP-9, which are involved in medial degradation, were detected already after 48 h (Atkins et al., 2014; Atkins, 2016). Similar evidences of dysregulations in gene expression and metalloproteinase concentration were also reported at the sites of highest $\left|\vec{WSS}\right|$ in BAV patients undergoing aortic resection, thus in presence of clinically-relevant aortopathy at the organ-scale length (Guzzardi et al., 2015).

Combining these previous evidences with the ones we obtained in the study herein presented we may speculate that $\vec{WSS}$ alterations precede the onset of aortopathy, that their short-term effect is detectable only at the cell level or at the microscale, and that the subsequent anatomical remodeling occurs only on the very long term. Should this speculation be correct, its practical implication would be that any follow-up study aimed at assessing the prognostic potential of fluid-dynamic alterations should be planned over a time-frame of decades.

## Limitations

The present study has some technical and clinical limitations that should be considered prior to drawing any strong conclusion based on our results.

As for the methods adopted to process 4D Flow, it is worth noticing that the definition of the ROI was based on manual tracings. As such, it was inherently affected by operator-dependent uncertainty. Such uncertainty impacts the definition of the local position of the aortic wall. Moreover, the ROI was defined only at the time-point with the highest signal-to-noise ratio, assumed as peak systole, and it was subsequently used to process velocity data at each systolic time-point of the dataset. The practical implication of this approach is that the real motion of the aortic wall was not accounted for. Of course, both the abovementioned aspects impact the reliability of computed $\left|\vec{WSS}\right|$ results, even though our values likely represent a better approximation of the friction actually exerted by the fluid on the vessel wall, as compared to the estimations obtained by alternative methods (Piatti et al., 2016).

Moreover, the spatial and temporal resolution of the datasets, even if compliant with the indications from the Consensum Statement (Dyverfeldt et al., 2015), inherently introduce limitations in the computation of space- and time-derivatives. The latter aspect in particular impacts the computation of OSI and of RRT.

From a clinical standpoint, the main limitation consists in the small number of subjects considered in the study. A larger number of healthy controls would allow for a more robust statistical distribution of data to be used as term of comparison for BAV data. Also, a larger cohort of BAV patients would allow to include relevant sub-populations with different fusion patterns and to verify the presence of fusion-specific features.

Follow-up was limited to a 3-year time-frame and did not include further 4D Flow acquisitions allowing for the evaluation of the changes in fluid dynamics occurring over time in the single patient. Extending the follow-up time-frame and performing such further acquisitions may yield relevant information on the role of fluid dynamics in the evolution toward the onset of the aortopathy or in the progression of the aortopathy itself following its onset.

## Ethics statement

Human subjects were enrolled and underwent clinical imaging acquisitions at John Radcliffe Hospital, Oxford, UK. The Institutional Review Board approved the study and informed consent was obtained from each participant.

## Author contributions

FP, SP, IN, and EV defined and implemented the algorithms to process 4D flow datasets and quantify the fluid-dynamic indexes of interest. MB performed the 4D flow acquisitions on the subjects enrolled in the study. FP and FS processed the datasets and performed the fluid-dynamic quantifications. MB, AD, and ML provided the clinical interpretation of results. AR provided the engineering interpretation of results. All authors contributed to conceiving the study and to writing the manuscript.

### Conflict of interest statement

The authors declare that the research was conducted in the absence of any commercial or financial relationships that could be construed as a potential conflict of interest.
