# Supplementary material for: 4D Flow Analysis of BAV-Related Fluid-Dynamic Alterations: Evidences of Wall Shear Stress Alterations in Absence of Clinically-Relevant Aortic Anatomical Remodeling
Source: Front Physiol. 2017 Jun 26;8:441. doi: 10.3389/fphys.2017.00441 (PMC5483483; doi:10.3389/fphys.2017.00441)
Supplement: Supplementary file 1 [file Table1.docx]

Supplementary Material

**4D Flow Analysis of BAV-related Fluid-dynamic Alterations: Evidences of Wall Shear Stress Alterations in Absence of Clinically-Relevant Aortic Anatomical Remodeling**

**Authors**: Filippo Piatti^1^, Francesco Sturla^1^, Malenka Bissell^2^, Selene Pirola^3^, Massimo Lombardi^4^, Igor Nesteruk^5^, Alessandro Della Corte^6^, Alberto Redaelli^1^, Emiliano Votta^1*^

^1^ Department of Electronics, Information and Bioengineering, Politecnico di Milano, Milan, Italy

^2^ Division of Cardiovascular Medicine, Radcliffe Department of Medicine, University of Oxford, Oxford, United Kingdom

^3^ Department of Chemical Engineering, Imperial College, London, United Kingdom

^4^ Multimodality Cardiac Imaging Section, IRCCS Policlinico San Donato, San Donato Milanese, Milan, Italy

^5^ Department of Free Boundary Flows, Institute of Hydromechanics, National Academy of Sciences of Ukraine, Kyiv, Ukraine

^6^ Department of Cardiothoracic and Respiratory Sciences, Second University of Naples, Naples, Italy

**Correspondence:**

Emiliano Votta

[emiliano.votta@polimi.it](mailto:emiliano.votta@polimi.it)

**Supplementary Table 1**. Definition of the directional Oscillatory Shear Index (OSI) along the axial component (OSI_Ax_) and circumferential component (OSI_Circ_) of the WSS vector, and of the time-averaged cross flow (CF) (Mohamied et al., 2017).

| Index | Definition |
| --- | --- |
| OSI_Ax_ [-] | $0.5\cdot\left( 1- \frac{\int_{0}^{T} \left\vert\vec{WSS}_{Ax} \right\vert dt}{\left\vert\int_{0}^{T} \vec{WSS}_{Ax}dt \right\vert} \right)$ |
| OSI_Circ_ [-] | $0.5\cdot\left( 1- \frac{\int_{0}^{T} \left\vert\vec{WSS}_{Circ} \right\vert dt}{\left\vert\int_{0}^{T} \vec{WSS}_{Circ}dt \right\vert} \right)$ |

**Supplementary Figure 1.** a) The 2D template detailed in Figure 3 of the manuscript is reported for the sake of clarity. b) OSI_Ax_ heat maps for HVs (represented as 10^th^, 50^th^ and 90^th^ percentiles) and for each BAV patient obtained through time-averaging over the systolic phase. For each patient-specific distribution of OSI_Ax_, the HV-relative heat maps were computed and color-coded accordingly: higher than 90^th^ percentile (red), lower than 10^th^ percentile (blue), between 90^th^ and 10^th^ percentiles (light gray, dark gray).

**Supplementary Figure 2.** a) The 2D template detailed in Figure 3 of the manuscript is reported for the sake of clarity. b) OSI_Circ_ heat maps for HVs (represented as 10^th^, 50^th^ and 90^th^ percentiles) and for each BAV patient obtained through time-averaging over the systolic phase. For each patient-specific distribution of OSI_Circ_, the HV-relative heat maps were computed and color-coded accordingly: higher than 90^th^ percentile (red), lower than 10^th^ percentile (blue), between 90^th^ and 10^th^ percentiles (light gray, dark gray).
