# Supplementary figures and images for: 4D Flow Analysis of BAV-Related Fluid-Dynamic Alterations: Evidences of Wall Shear Stress Alterations in Absence of Clinically-Relevant Aortic Anatomical Remodeling
Source: Front Physiol. 2017 Jun 26;8:441. doi: 10.3389/fphys.2017.00441 (PMC5483483; doi:10.3389/fphys.2017.00441)

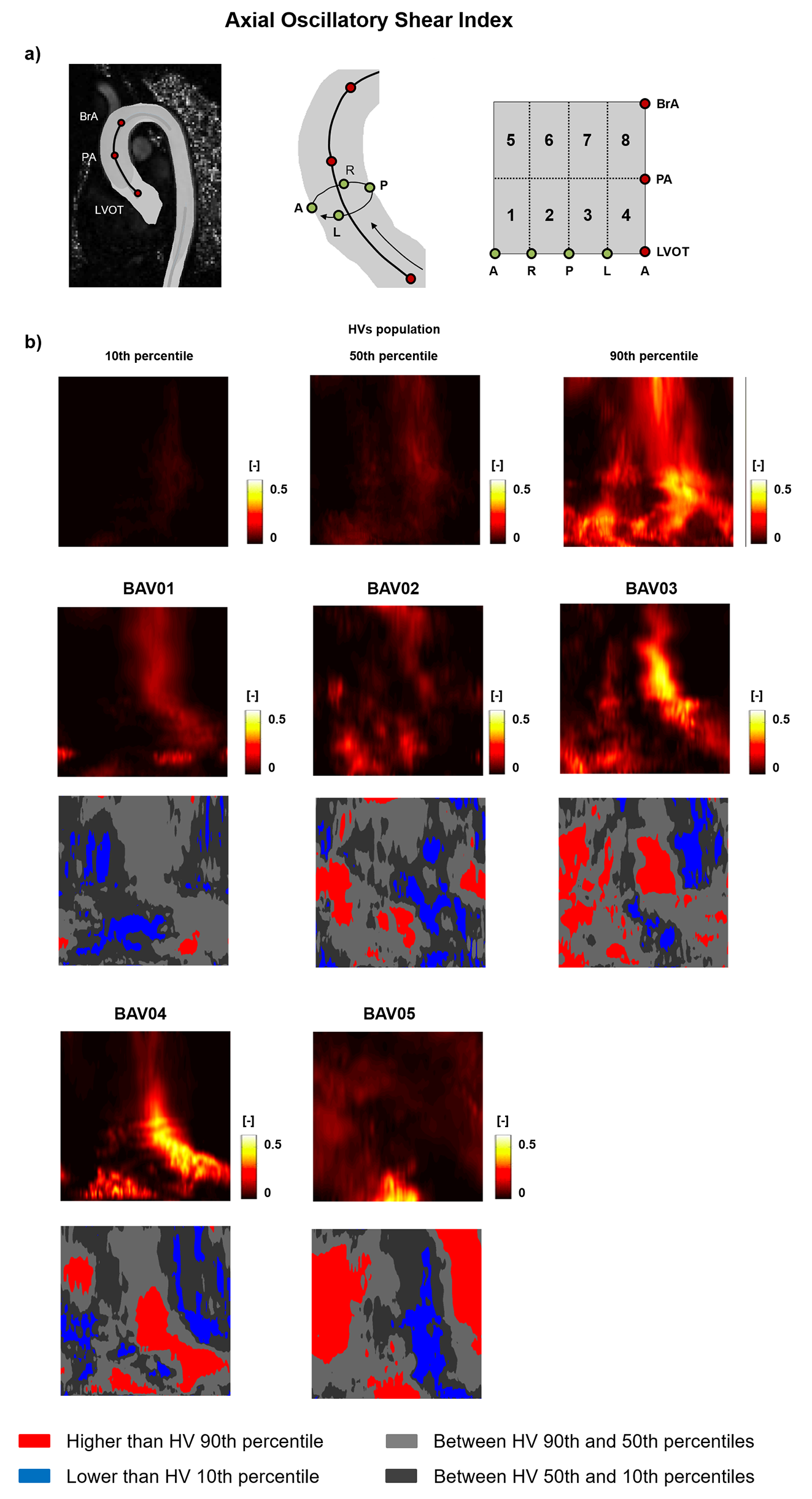

Supplement: Supplementary file 2 [file Image1.TIF]

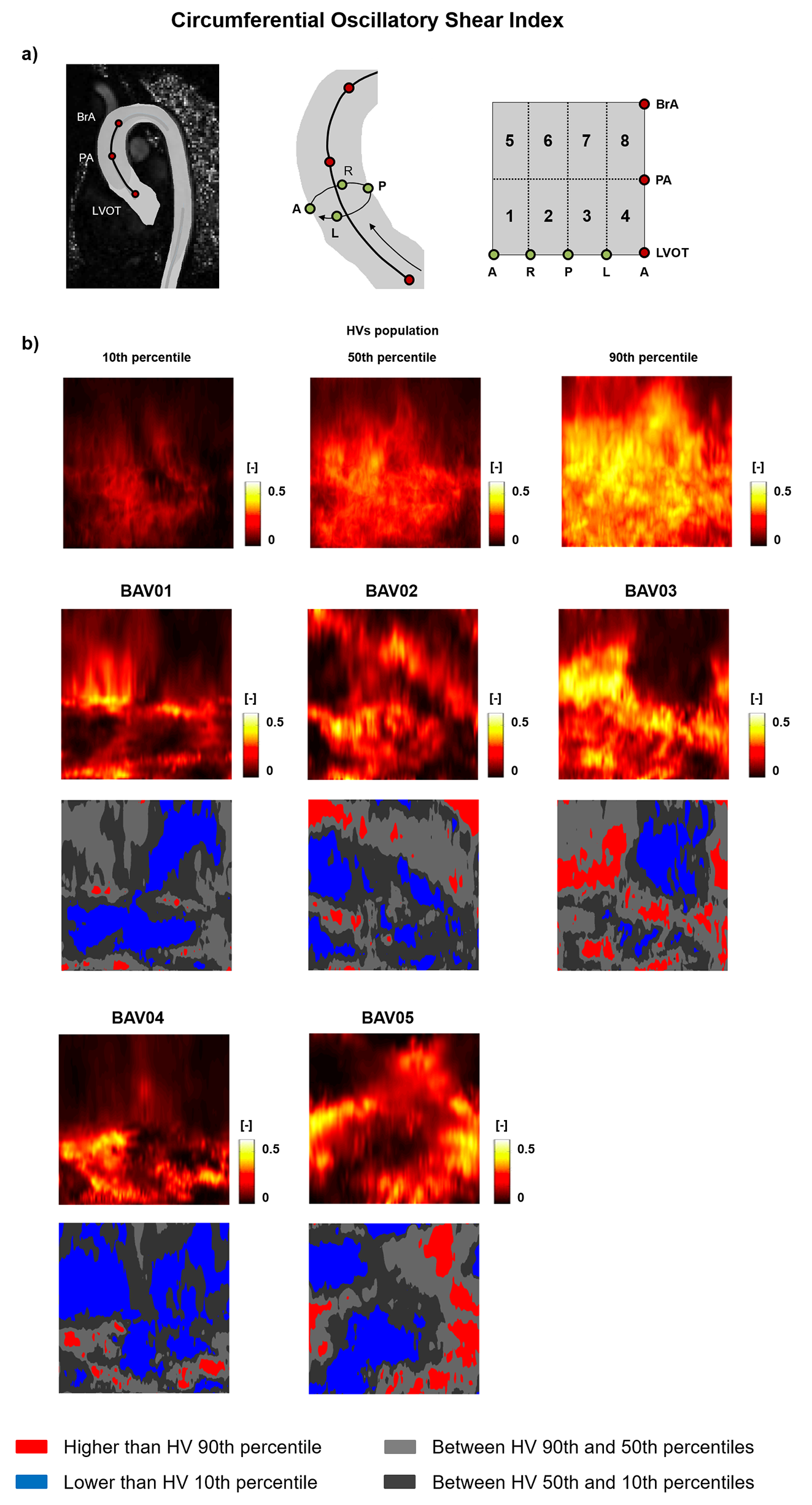

Supplement: Supplementary file 3 [file Image2.TIF]
